# Rethinking foreign language boredom in AI-assisted learning: the hidden roles of growth mindset, AI literacy, and AI self-efficacy

**DOI:** 10.3389/fpsyg.2026.1876813

**Published:** 2026-06-19

**Authors:** Wan Qin, Yanyan Chen, Hui Wang, Qiangwei Lu

**Affiliations:** 1 School of Education, Huaibei Institute of Technology, Huaibei, China; 2 Faculty of Education, Languages & Arts, INTI International University, Nilai, Malaysia

**Keywords:** growth mindset, AI literacy, AI self-efficacy, EFL boredom, EFL learning

## Abstract

**Introduction:**

With the rapid integration of artificial intelligence (AI) into English as a foreign language (EFL) learning, learners’ emotional experiences have attracted increasing attention. Among these emotions, EFL boredom, a persistent negative emotion, can substantially undermine motivation, classroom engagement, and learning outcomes. Although previous studies suggest that growth mindset may alleviate negative emotions in EFL learning, its influence on EFL boredom in AI-assisted learning environments remains underexplored. Meanwhile, AI literacy and AI self-efficacy may also play important roles. Therefore, Drawing on the frameworks of Social Cognitive Theory (SCT) and Control-value Theory(CVT), this study examined the relationships among growth mindset, AI literacy, AI self-efficacy, and EFL boredom and further constructed a sequential mediation model.

**Methods:**

A time-lagged design was adopted, involving three waves of data collection among 536 university first-year students from two higher education institutions in eastern China. Growth mindset was measured in Phase 1, AI literacy and AI self-efficacy in Phase 2, and EFL boredom in Phase 3, with two-month intervals between phases. Data were collected using validated scales. SPSS 26.0 and AMOS 21.0 were employed for descriptive statistics, correlation analysis, structural equation modeling (SEM), and Bootstrap mediation analyses.

**Results:**

The results showed that growth mindset positively predicted AI literacy and AI self-efficacy. Both AI literacy and AI self-efficacy negatively predicted EFL boredom, while AI literacy further positively predicted AI self-efficacy. However, growth mindset did not directly predict EFL boredom. In addition, AI literacy and AI self-efficacy exerted significant independent and sequential mediating effects in the relationship between growth mindset and EFL boredom.

**Discussion:**

The findings suggest that growth mindset alleviates EFL boredom indirectly through AI-related competencies and beliefs rather than through a direct pathway. The study highlights the important roles of AI literacy and AI self-efficacy in shaping emotional experiences in AI-assisted EFL learning and extends the application of growth mindset theory to AI-enhanced language education.

## 1 Introduction

English as a foreign language (EFL) learning is a process laden with positive and negative emotions (Boudreau et al., 2018; Derakhshan et al., 2022). Among these emotions, boredom has recently emerged as a salient yet underexplored negative affective factor in EFL contexts. Within the framework of the control-value theory (CVT) (Pekrun, 2006), boredom arises when learners attach little value to the achievement activity and perceive low control over them. As a typical negative mental state characterized by a lack of interest in learning tasks (Pawlak et al., 2020), EFL learners may experience boredom when they struggle to effectively engage with learning tasks and perceive insufficient competence. It has been shown to have a pernicious impact on leaners’ EFL motivation (Nikitina et al., 2024) and self-regulated learning strategies (Luo and Wang, 2023), and classroom engagement (Zhao et al., 2025), thereby ultimately deactivating their L2 achievement (Huang and Zhang, 2025; Li et al., 2025; Li and Yang, 2025; Zhang and Li, 2025; Lu et al., 2026). Given its crucially negative role in EFL development, it is necessary to identify the cognitive and environmental variables underlying EFL boredom.

Growth mindset is conceptualized as a malleable trait, involving learners’ belief that their abilities and competence can be developed through consistent effort and personal determination (Dweck, 2006). Within EFL learning contexts, in contrast to a fixed mindset, which assumes abilities are static and innate, learners with a growth mindset tend to regulate their learning strategies and take more positive emotions when facing challenges (Lou and Noels, 2019). While fixed mindset, attributing low value to effort, is naturally related to negative emotional tendencies, growth mindset has also been found to be negatively associated with these tendencies (Lou and Zarrinabadi, 2022), which is more likely to adapt to emotional outcomes and environmental intervention. In light of this, understanding the adaptive effect of growth mindset on learners’ negative emotional tendencies (e.g., boredom) is essential. Moreover, from the perspective of CVT, characterized as a positive belief, growth mindset can serve as a personality antecedent to achievement emotions by influencing learners’ control-value appraisals. In terms of how growth mindset affects EFL emotions, emerging evidence suggests that it may enhance positive emotions (e.g., enjoyment) and alleviate negative emotions (e.g., boredom) (Derakhshan et al., 2022; Lou et al., 2025; Wang et al., 2021; Farsani and Seyedshoja, 2024). However, despite the promising role of growth mindset in shaping EFL learners’ emotional outcomes such as boredom, the underlying mechanisms through which growth mindset mitigates boredom remain insufficiently understood. In particular, the rapidly advancing artificial intelligence (AI) technologies may introduce new variables in EFL learning environments (Liu, 2026), highlighting the need to examine how growth mindset shape boredom in AI-assisted EFL learning.

Situated against the backdrop of AI, the learning mode of EFL learners have been fundamentally reshaped (Barrot, 2023). According to social cognitive theory (SCT) (Bandura, 1986), learners’ learning processes are dynamically and reciprocally interacted by their cognition, behavior, and the environment. Amid a new environment created by AI, the process of learning and development has also undergone drastic transformation. AI tools have been confirmed to enhance EFL learners’ emotional, cognitive, and behavioral states, and therefore mobilize their enthusiasm and motivation for language learning (Derakhshan, 2025; Wang et al., 2025). However, compared to the cognitive and behavioral benefits of AI-assisted learning, learners’ affective states in the context of AI have garnered less attention (Liu, 2026). This issue is particularly remarkable as AI-assisted learning have introduced unprecedented social environment variables such as the way of EFL learner understand, evaluate, and interact with AI, which may mediate EFL learners’ situational control-value appraisals, therefore influencing their achievement emotions (Pekrun, 2006). Among limited current studies, while prior studies have shown remarkable affective advantages of AI-assisted learning in the context of EFL learning, particularly in increasing enjoyment and decreasing anxiety (Liu et al., 2024; Zhao, 2025; Shen and Tao, 2025; Zhang and Liu, 2025), it is still not clear whether EFL boredom can be reduced or catalyzed.

AI literacy and AI self-efficacy are two significant constructs in the landscape of AI-assisted EFL learning. AI literacy is closely related to learners’ knowledge of how to understand, apply and evaluate AI tools as well as their critical awareness of AI ethics (Ng et al., 2021; Ng et al., 2024). Although it is found that AI can contribute to the improvement of language learning output, it simultaneously raises concerns about over-reliance and the lack of critical thinking (Wang et al., 2024), which is largely associated with the short of AI literacy (Pérez-Paredes et al., 2025). In the realm of EFL learning, learners with higher AI literacy are better equipped to utilize AI tools effectively and critically and navigate AI-assisted learning environments, thereby fostering more adaptive EFL learning attitudes and emotional outcomes (Kong et al., 2025; Zou et al., 2026). However, research investigating the relation between AI literacy and EFL emotions mainly focuses on positive ones (e.g., Ng et al., 2024), particularly enjoyment (e.g., Fan and Zhang, 2024). Placing AI literacy within the CVT, this study posits that it may strengthen learners’ perceived control over AI-assisted learning tasks and enhance the perceived value of these activities, which may potentially reduce boredom. Nevertheless, this possibility remains underexplored in existing EFL research.

AI self-efficacy, serves as another pivotal construct in the domain of AI, referring to learners’ perception in their ability to apply and interact with AI tools to fulfill learning tasks (Wang and Chuang, 2024). SCT posits that efficacy beliefs are developed through knowledge acquisition and mastery experiences (Bandura, 1977). As learners acquire more AI-related knowledge, they are likely to develop stronger confidence in their ability to use AI tools effectively. This interpretation has been supported by prior findings suggesting that AI literacy serves as an important antecedent of AI self-efficacy (Bewersdorff et al., 2025; Zhang et al., 2025). In addition, according to Zhang et al. (2025), AI literacy is positively predictive to the enhancement of AI self-efficacy, consequently diminishing anxiety and promoting EFL learning outcomes, which indicates that AI literacy and AI self-efficacy may sequentially mediate EFL learners’ emotions. However, there is limited investigation into how these two constructs relate to each other and function on EFL emotions such as boredom.

Despite the calls for further understanding into the mechanism of growth mindset in influencing EFL learners’ boredom in AI-mediated learning contexts, to the best of our knowledge, few studies delve into the potential mediating role of AI literacy and AI self-efficacy in this process. Based on these research gaps, this study draws on the frameworks of SCT and CVT and adopts a time-lagging design to answer the following two research questions: (1) What are the relationships between growth mindset, AI literacy and AI self-efficacy? (2) How these factors relate to each other and contribute to EFL boredom? This study proposes a hypothetically sequential mediating model that will be further discussed in methodology. In light of it, the present study has several contributions. First, it extends growth mindset research by examining its relationship with EFL boredom in AI-mediated EFL learning, thereby enriching our understanding of growth mindset in emerging AI-assisted language learning contexts (Lou and Zarrinabadi, 2022; Mohammadzadeh Mohammadabadi, 2025; Zarrinabadi and Lou, 2022). Second, by integrating SCT and CVT, it provides a more comprehensive theoretical explanation of how growth mindset, AI literacy, AI self-efficacy, and EFL boredom are interconnected. Third, it empirically examines the previously underexplored sequential mediating roles of AI literacy and AI self-efficacy in linking growth mindset to EFL boredom. Fourth, unlike most prior studies employing cross-sectional designs, it adopts a time-lagged design to capture the temporal dynamics among these variables. Finally, it offers pedagogical insights into fostering learners’ AI-related competencies to reduce EFL boredom and promote more engaging learning experiences. Finally, it will offer pedagogical insights into how to foster EFL learners’ AI-related competencies to mitigate EFL boredom and promote more engaging EFL learning experiences.

## 2 Literature review

### 2.1 Growth mindset and EFL boredom in AI-assisted EFL learning

Growth mindset, proposed by Dweck (2006), refers to a positive belief that individuals’ abilities and intelligence can be continuously developed through sustained effort, strategic adjustment, and accumulated experience. Growth mindset has been widely recognized as an important psychological variable influencing learning motivation, strategy use, emotional experiences, and academic achievement (Yeager et al., 2019; Zhang et al., 2024). Research has shown that learners with a growth mindset are more likely to perceive difficult tasks as opportunities for development rather than as indicators of insufficient ability, and are therefore more capable of maintaining learning engagement and persistence in complex learning environments (e.g., Yeager and Dweck, 2012).

In the field of EFL learning, growth mindset not only influences learners’ cognition and behaviors but also further shapes their emotional experiences (Wei et al., 2026). Compared with learners holding a fixed mindset, who are more likely to withdraw when encountering errors or difficulties, learners with a growth mindset tend to maintain a more positive attitude toward exploration and continuous effort (Mueller and Dweck, 1998). Previous studies have indicated that growth mindset can not only enhance learning motivation and classroom engagement but also alleviate negative emotions such as anxiety and frustration (e.g., Wang et al., 2021; Derakhshan et al., 2022; Wei et al., 2026). Meanwhile, learners with a growth mindset are more likely to adopt deep-processing and active knowledge-construction strategies, such as proactively seeking opportunities for language input and output and continuously adjusting their learning approaches based on feedback, rather than relying on rote memorization or surface-level practice (Dweck, 2017; Xu et al., 2025).

In recent years, with the gradual integration of AI into EFL learning contexts, the role of growth mindset in AI-assisted EFL learning has become increasingly important. AI-assisted learning not only requires learners to possess traditional language learning abilities but also demands that they be able to understand, utilize, and evaluate AI-generated information and feedback (Barrot, 2023). In this context, learners with a growth mindset are generally more willing to actively experiment with new technologies, explore the learning functions of AI tools, and maintain stronger learning resilience and adaptability when facing technological challenges (Huang et al., 2025). Therefore, in AI-assisted EFL learning environments, growth mindset is not only an important psychological trait but has also gradually become a key factor influencing learners’ technological adaptation, learning strategies, and the development of AI-related competencies (Huang et al., 2025).

Meanwhile, academic emotions primarily arise from learners’ subjective evaluations of their sense of *control* and *value* regarding learning tasks (Pekrun, 2006). As a typical low-arousal negative academic emotion, EFL boredom often emerges when learners perceive learning tasks as meaningless, insufficiently challenging, or difficult to control (Li and Li, 2024). In AI-assisted EFL learning environments, even if learners possess positive beliefs about their abilities, their sense of control over learning activities may still remain low if they are unable to effectively understand or use AI tools, which may consequently lead to EFL boredom. Therefore, whether growth mindset can directly reduce EFL boredom in AI-assisted EFL learning contexts remains to be further verified. Based on the above discussion, the first hypothesis of the present study could be formulated as follows:

*H1*: Growth mindset negatively predicts EFL boredom.

### 2.2 AI literacy as a mediator between growth mindset and EFL boredom

AI literacy is generally defined as individuals’ comprehensive ability to recognize, understand, use, evaluate, and ethically engage with AI technologies (Long and Magerko, 2020; Al-Abdullatif, 2025). In recent years, researchers have further conceptualized AI literacy as a multidimensional construct encompassing AI conceptual understanding, tool operation skills, output evaluation ability, ethical awareness, and collaboration competence between human and AI (Wang et al., 2023; Celik et al., 2025). Compared with traditional digital literacy, AI literacy places greater emphasis on learners’ abilities to understand, critically evaluate, and collaboratively interact with AI-generated content (Ng et al., 2021; Ng et al., 2024). As AI technologies are increasingly integrated into educational contexts, AI literacy has been recognized as a crucial competence for learners’ adaptation to AI-assisted learning environments and has been found to significantly influence learning engagement, learning experiences, and learning outcomes.

In the field of EFL learning, AI literacy involves not only technical operational skills but also learners’ abilities to select appropriate AI tools according to learning objectives, evaluate AI-generated outputs, and effectively utilize AI feedback (Gu and Ericson, 2025; Dong et al., 2025). Previous studies have shown that learners with higher levels of AI literacy are generally more capable of proactively utilizing AI resources for autonomous learning and demonstrate stronger learning engagement and greater perceived control during human-and-AI interactions (Liu, 2026). In contrast, learners with lower levels of AI literacy may experience confusion and negative emotions during the learning process because they are unable to effectively interpret AI feedback or properly use AI tools. These findings suggest that, within AI-assisted EFL learning contexts, AI literacy functions not only as a technological competence but also as an important learning resource.

Existing research has indicated that learners with a growth mindset are more willing to actively engage with new technologies, experiment with innovative learning approaches, and maintain higher levels of openness and adaptability during technology-assisted learning processes (e.g., Huang et al., 2025). Growth mindset emphasizes the malleability of ability, suggesting that learners believe their competencies can be continuously developed through sustained effort and accumulated experience (Dweck, 2006). Therefore, compared with learners holding a fixed mindset, learners with a growth mindset are more likely to persist in exploration and active experimentation when encountering difficulties in AI-assisted learning, thereby gradually accumulating AI-related knowledge and technological experience. From the perspective of SCT, learners’ cognitive beliefs influence their behavioral engagement and interactions with the environment (Bandura, 1986). In AI-assisted EFL learning contexts, growth mindset, as a positive cognitive belief, may encourage learners to participate more actively in AI-assisted learning activities and continuously enhance their AI literacy through ongoing human-and-AI interactions. At the same time, higher levels of AI literacy may enable learners to better understand AI feedback, critically filter AI-generated content, and enhance their learning autonomy and task engagement (Liu, 2026). Consequently, when learners are able to exert greater control over learning tasks and perceive stronger value in learning activities, their negative emotions, like boredom, are likely to decrease accordingly (Pekrun, 2006). Therefore, AI literacy may serve as an important mediating variable in the relationship between growth mindset and EFL boredom. Based on the above discussion, the second hypothesis of the present study could be formulated as follows:

*H2*: Growth mindset positively predicts AI literacy, AI literacy negatively predicts EFL boredom, and AI literacy mediates the relationship between growth mindset and EFL boredom.

### 2.3 AI self-efficacy as a mediator between growth mindset and EFL boredom

AI self-efficacy refers to individuals’ subjective judgments regarding their ability to effectively understand, operate, and utilize AI technologies to accomplish relevant tasks (Wang and Chuang, 2024). Its theoretical foundation is derived from Bandura’s (1977) self-efficacy theory, which posits that individuals’ beliefs about their own capabilities significantly influence their behavioral choices, effort expenditure, task persistence, and emotional responses. With the increasing integration of AI technologies into educational settings, AI self-efficacy has been regarded as an important psychological factor influencing learners’ technological adaptation, learning engagement, and learning experiences.

In AI-assisted learning environments, high levels of AI self-efficacy can strengthen learners’ technological confidence when facing complex or unfamiliar AI-related tasks, making them more willing to adopt strategies of experimentation and adjustment rather than avoidance or withdrawal (Wang and Chuang, 2024). Previous studies have suggested that learners with a growth mindset are generally more likely to believe that they can gradually master new knowledge and skills through sustained effort, thereby developing more positive judgments of their technological capabilities and stronger learning confidence (e.g., Huang et al., 2025). Growth mindset emphasizes the developmental nature of ability (Dweck, 2006), and this positive cognitive belief may help learners maintain higher levels of persistence and adaptability when encountering technological challenges in AI-assisted learning. Since learners’ cognitive beliefs further influence their behavioral engagement and learning outcomes (Bandura, 1986), learners with a growth mindset, compared with those holding a fixed mindset, are more likely to actively participate in AI-assisted learning activities and gradually develop higher levels of AI self-efficacy through continuous human-and-AI interactions and the accumulation of successful experiences.

Meanwhile, AI self-efficacy may further shape learners’ emotional experiences in AI-assisted EFL learning contexts. Bandura (1986, 2003) argued that higher levels of self-efficacy can enhance learners’ sense of competence regarding learning tasks, enabling them to demonstrate greater behavioral engagement, strategy use, and task persistence when facing complex or challenging learning activities, while simultaneously reducing negative emotional experiences. In addition, when learners perceive a stronger sense of control over learning tasks, negative emotions such as boredom are likely to decrease accordingly (Pekrun, 2006). In AI-assisted EFL learning contexts, when learners believe that they are capable of effectively using AI tools to accomplish learning tasks, their technological stress, uncertainty, and helplessness may be reduced, thereby alleviating EFL boredom. Therefore, AI self-efficacy may serve as a mediating variable in the relationship between growth mindset and EFL boredom. Based on the above discussion, the third hypothesis of the present study could be formulated as follows:

*H3*: Growth mindset positively predicts AI self-efficacy, AI self-efficacy negatively predicts EFL boredom, and AI self-efficacy mediates the relationship between growth mindset and EFL boredom.

### 2.4 The sequential mediating roles of AI literacy and AI self-efficacy

Although the effects of AI literacy and AI self-efficacy on learning behaviors and emotional experiences have been separately examined, the relationship between these two constructs remains insufficiently explored. Bewersdorff et al. (2025) suggested that AI literacy may serve as an important prerequisite for the development of AI self-efficacy. Compared with mere technological knowledge acquisition, AI self-efficacy places greater emphasis on learners’ subjective judgments regarding their ability to effectively use AI technologies, and such judgments are often grounded in accumulated knowledge and practical experience. Self-efficacy does not develop in isolation; rather, it gradually evolves through continuous interactions between individuals and their environments, involving knowledge acquisition, behavioral practice, and the accumulation of successful experiences (Bandura, 1986). Therefore, a progressive relationship may exist between AI literacy and AI self-efficacy.

Specifically, learners with higher levels of AI literacy are generally better able to understand the operational logic, usage methods, and feedback mechanisms of AI tools and are more likely to accumulate positive human-and-AI interaction experiences during AI-assisted learning processes (Bewersdorff et al., 2025). Meanwhile, learners with higher AI literacy are often more capable of effectively filtering AI-generated content, adjusting learning strategies, and resolving technical problems encountered during learning activities, thereby gaining more successful experiences in AI-assisted learning contexts. According to Bandura (1977), mastery experiences constitute one of the most important sources of self-efficacy. Therefore, when learners are able to competently understand and utilize AI tools, their confidence in their ability to use AI technologies is likely to increase accordingly, resulting in higher levels of AI self-efficacy. Previous studies have also indicated that learners’ technological knowledge and digital competence can further enhance their technological confidence and adaptability (e.g., Ng et al., 2021). These findings suggest that AI literacy is not only a technological competence but may also serve as an important foundation for the development of AI self-efficacy.

**Figure 1:**
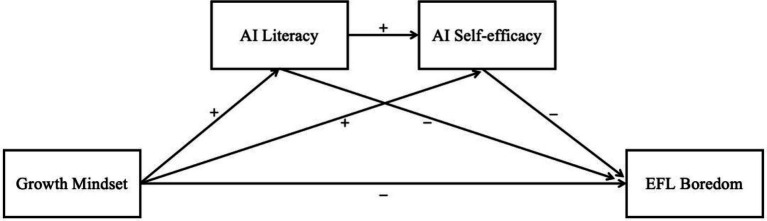
Research hypothesis model.

As discussed above, growth mindset, as a positive belief about the malleability of ability, may enhance learners’ willingness to actively engage in AI-assisted learning activities and gradually improve their AI literacy through continuous human-and-AI interactions and practical experiences. Meanwhile, when learners become more capable of effectively understanding and utilizing AI tools, their AI self-efficacy is also likely to increase accordingly, which may further enhance their sense of control over learning and classroom engagement, ultimately reducing EFL boredom. Therefore, AI literacy and AI self-efficacy may exhibit a progressive and dynamic relationship and jointly function as sequential mediators in the relationship between growth mindset and EFL boredom. Based on the above discussion, the last hypothesis of the present study could be formulated as follows and the proposed research hypothesis model can be seen in Figure 1:

*H4*: AI literacy positively predicts AI self-efficacy, and AI literacy together with AI self-efficacy jointly plays a sequential mediating role in the relationship between growth mindset and EFL boredom.

## 3 Methodology

### 3.1 Participants

After obtaining consent from the participating universities, teachers, and students, the researchers recruited participants through convenience sampling from two universities located in a city in eastern China, including one normal university and one institute of technology. The participants were all first-year students, and majored in a range of disciplines, such as Chinese studies, marketing, preschool education, automation, economics, mathematics, and computer science etc., covering fields in science and humanities. A total of 553 questionnaires were collected. After excluding 17 invalid responses due to duplicate submissions, 536 valid questionnaires were retained, yielding an effective response rate of 96.92%. Among the valid participants, 45.38% were male and 54.62% were female, with ages ranging from 20 to 22 years. The participants had studied English for approximately 15 years and engaged in more than six hours of EFL learning per week. Their English proficiency level corresponded to a vocabulary size of approximately 3,200 words, enabling them to read English novels of moderate difficulty and conduct simple daily conversations. All participants used the same nationally standardized College English textbook, *New Horizon College English 2*. The curriculum standards and teaching syllabus were uniformly formulated and implemented at the national level.

During the data collection period, all participants attended four English classes per week, with each class lasting 45 min. In addition, because the College English curriculum standards require learners to develop the ability to utilize AI for relevant learning tasks, all participants in this study had experience using generative AI tools in the process of college English learning. These AI applications were primarily used for EFL learning tasks such as writing assistance, translation support, and information retrieval. The AI tools used included, but were not limited to, conversational AI models such as DeepSeek, ChatGPT, and Doubao. Participants demonstrated varying levels of proficiency in AI tool use, with most exhibiting basic to intermediate competence. Specifically, they were generally able to understand and employ basic AI prompts to accomplish fundamental text generation and revision tasks. In classroom teaching contexts, EFL teachers employed online learning-support platforms integrated with AI tools to facilitate specific in-class learning activities. Overall, AI tools were mainly applied in activities such as English writing practice, Chinese-English and English-Chinese translation exercises, comprehension and translation of complex sentence structures, and reading comprehension support.

In this study, although demographic characteristics and institutional differences may have constituted potential sources of confounding, several important background variables (e.g., prior English proficiency and socioeconomic background) were not collected during the data collection process and therefore could not be included as covariates in the analysis. In addition, given the limited number of participating institutions, the study did not employ multilevel modeling or cluster-robust standard errors to account for the nested structure of the data. To partially reduce contextual heterogeneity, all participating institutions followed nationally unified curriculum standards, adopted the same textbook (*New Horizon College English 2*), and implemented a consistent teaching syllabus. Nevertheless, these control measures may not have been sufficient to fully eliminate residual confounding effects or completely address potential clustering dependency at the institutional level.

### 3.2 Research instruments

Data in this study were collected through questionnaires. The questionnaire consisted of two sections. The first section gathered participants’ demographic information, specifically gender. The second section included four Likert-scale instruments: Growth Mindset Scale, AI Literacy Scale, AI Self-Efficacy Scale, and Foreign Language Boredom Scale. All instruments employed a five-point Likert scale. In addition, to facilitate participants’ comprehension and enhance the reliability of the data, all questionnaire items were presented in Chinese. The results for all scales indicate satisfactory reliability and validity according to relevant fitting cutoff criteria (Wen et al., 2004).

#### 3.2.1 Growth mindset scale

Growth mindset was measured using the Growth Mindset Scale developed and validated by Sigmundsson and Haga (2024). The scale consists of eight items (Items 2–9), including statements such as “I know that with effort I can improve my skills and knowledge” and “Effort makes me stronger.” The scale demonstrated satisfactory internal consistency reliability (Cronbach’s *α* = 0.911) and construct validity, with acceptable model fit indices (χ^2^/df = 2.610 < 3; CFI = 0.927 ≥ 0.90; TLI = 0.913 ≥ 0.90; SRMR = 0.048 ≤ 0.08; RMSEA = 0.043 ≤ 0.08).

#### 3.2.2 AI literacy scale

AI literacy was measured using the AI Literacy Scale developed and validated by Wang et al. (2023). The scale consists of 12 items (Items 10–21) across four dimensions: AI Awareness, AI Usage, AI Evaluation, and AI Ethics. Example items include “I can distinguish between smart devices and non-smart devices” (AI Awareness), “I can use AI applications or products to help me with my daily work” (AI Usage), “I can choose the most appropriate AI application or product from a variety for a particular task” (AI Evaluation), and “I am always alert to the abuse of AI technology” (AI Ethics). The scale demonstrated satisfactory internal consistency reliability (Cronbach’s *α* = 0.923) and construct validity, with acceptable model fit indices (χ^2^/df = 1.973 < 3; CFI = 0.974 ≥ 0.90; TLI = 0.946 ≥ 0.90; SRMR = 0.059 ≤ 0.08; RMSEA = 0.022 ≤ 0.08).

#### 3.2.3 AI self-efficacy scale

AI self-efficacy was measured using the AI Self-Efficacy Scale developed and validated by Wang and Chuang (2024). The scale comprises 22 items (Items 22–43) across four dimensions: Assistance, Anthropomorphic Interaction, Comfort with AI, and Technological Skills. Example items include “I find that AI technologies/products are helpful for learning” (Assistance), “I think the interactive process of AI technologies/products is very vivid, just like chatting with a real person” (Anthropomorphic Interaction), “When interacting with AI technologies/products, I feel very relaxed” (Comfort with AI), and “AI technologies/products jargon does not baffle me” (Technological Skills). The scale demonstrated satisfactory internal consistency reliability (Cronbach’s α = 0.940) and construct validity, with acceptable model fit indices (χ^2^/df = 2.734 < 3; CFI = 0.934 ≥ 0.90; TLI = 0.957 ≥ 0.90; SRMR = 0.041 ≤ 0.08; RMSEA = 0.028 ≤ 0.08).

#### 3.2.4 Foreign language classroom boredom scale

EFL boredom was measured using the Foreign Language Classroom Boredom Scale developed and validated by Li et al. (2023), which is a subscale of the Foreign Language Learning Boredom Scale (Li et al., 2023). The scale contains eight items (Items 44–51), including statements such as “The English class bores me” and “Time is dragging on in English class.” The scale demonstrated satisfactory internal consistency reliability (Cronbach’s *α* = 0.972) and construct validity, with acceptable model fit indices (χ^2^/df = 2.593 < 3; CFI = 0.919 ≥ 0.90; TLI = 0.949 ≥ 0.90; SRMR = 0.033 ≤ 0.08; RMSEA = 0.021 ≤ 0.08).

### 3.3 Data collection

This study employed a time-lagged research design for data collection and conducted three waves of questionnaire surveys in order to reduce the potential influence of common method bias to some extent (Yu et al., 2021).

The interval between each wave of data collection was two months. Each survey was administered on a class-by-class basis in classroom settings, with questionnaires distributed and collected on-site through an online survey platform. To ensure longitudinal matching across the three waves of data collection, all participants were required to provide the last four digits of their student ID numbers as a unique identification code.

In the first phase (January 2026), the Growth Mindset Scale was administered. In the second phase (March 2026), the AI Literacy Scale and the AI Self-Efficacy Scale were administered. In the third phase (May 2026), the Foreign Language Learning Boredom Scale was administered.

During data processing, only questionnaires that were successfully matched across all three waves and fully completed were retained for analysis. Responses that could not be matched longitudinally or contained missing data were excluded. Ultimately, 536 valid samples (*N* = 536) were obtained, all of which consisted of participants who had completed all three phases of the survey with valid responses at each stage.

### 3.4 Data analysis

Prior to data analysis, an *a priori* power analysis was conducted using G*Power 3.1 to evaluate the adequacy of the sample size. Following Cohen (2013), a medium effect size (f^2^ = 0.15), a significance level of α = 0.05, a statistical power of 0.95, and three predictors were specified. The results indicated that a minimum sample size of 119 participants was required. Therefore, the final sample of 536 participants substantially exceeded the recommended threshold, indicating sufficient statistical power for testing the hypothesized relationships and mediation effects. Then, SPSS 26.0 was employed to conduct descriptive and correlation analyses in order to examine the basic relationships among growth mindset, AI literacy, AI self-efficacy, and EFL boredom. Next, AMOS 21.0 was used to construct a structural equation modeling (SEM) framework and simultaneously establish the structural paths among growth mindset, AI literacy, AI self-efficacy, and EFL boredom. Path analysis was then conducted to test the hypotheses proposed in this study. Besides, mediation analyses were conducted to examine the mediating roles of AI literacy and AI self-efficacy, with 5,000 bootstrap resamples performed to test the significance of the mediation effects. Finally, indices such as χ^2^/df, CFI, TLI, RMSEA, and SRMR would be used to evaluate model fit in the SEM analysis. Their cutoff criteria are as follows: χ^2^/df < 3.000, CFI ≥ 0.900, TLI ≥ 0.900, RMSEA ≤ 0.080 and SRMR ≤ 0.080 (Wen et al., 2004).

## 4 Findings

### 4.1 Overall description of growth mindset, AI literacy, AI self-efficacy, and EFL boredom

Descriptive statistics and Pearson correlation analyses conducted using SPSS 26.0 indicated that (see Table 1) growth mindset was positively correlated with both AI literacy and AI self-efficacy, while it was negatively correlated with EFL boredom. AI literacy was positively correlated with AI self-efficacy and negatively correlated with EFL boredom. In addition, AI self-efficacy was negatively correlated with EFL boredom.

**Table 1:** Descriptive statistics and correlation analysis (*N* = 536).

Items	Mean	SD	Growth mindset	AI literacy	AI self-efficacy	EFL boredom
Growth mindset	4.039	0.586	—			
AI literacy	3.725	0.571	0.391**	—		
AI self-efficacy	3.535	0.520	0.427**	0.657**	—	
EFL boredom	2.244	0.933	−0.391**	−0.237**	−0.104**	—

**p < 0.01.

### 4.2 Path analysis results in SEM

Inferential statistical analyses of the relationships and path coefficients among the variables were conducted using AMOS 21.0. The indications of results using standardized path coefficient (*β*) and *p* are as follows:

1. Growth mindset did not directly predict EFL boredom (β = −0.099, *p* > 0.01);
2. Growth mindset positively predicted AI literacy and AI self-efficacy (β = 0.535, *p* < 0.01; β = 0.162, *p* < 0.01, respectively), indicating that growth mindset contributes significantly to the enhancement of learners’ AI literacy and AI self-efficacy, particularly with respect to AI literacy;
3. AI literacy and AI self-efficacy negatively predicted EFL boredom (β = −0.402, *p* < 0.01; β = −0.550, *p* < 0.01, respectively), both reflecting relatively strong effect sizes, thereby suggesting that AI literacy and AI self-efficacy substantially contribute to the reduction of learners’ EFL boredom;
4. AI literacy positively predicted AI self-efficacy (β = 0.661, *p* < 0.01), reflecting a relatively strong effect size, thereby suggesting that AI literacy substantially contributes to the development of learners’ AI self-efficacy.

Based on the significance of the structural paths, model fit indices were further examined for the hypothesized model (see Table 2). The results showed that χ^2^/df was 1.933, while the values of CFI and TLI were 0.947 and 0.921, respectively, both exceeding the recommended threshold of 0.900. In addition, the RMSEA and SRMR values were 0.067 and 0.032, respectively, both below the recommended cutoff value of 0.080. These results indicate that the proposed model demonstrated a satisfactory fit and an overall acceptable level of model adequacy.

**Table 2:** Summary of model fit indicators for the proposed model.

Model fit indices	χ2/df	CFI	TLI	RMSEA	SRMR
Recommended cutoff	<3.000	≥0.900	≥0.900	≤0.080	≤0.080
Proposed model	1.933	0.947	0.921	0.067	0.032

Estimation method: maximum likelihood (ML) estimation.

### 4.3 Mediation analysis of AI literacy and AI self-efficacy between growth mindset and EFL boredom

To examine the independent and sequential mediating effects of AI literacy and AI self-efficacy in the relationship between growth mindset and EFL boredom, this study employed the bias-corrected Bootstrap method (5,000 resamples with a 95% confidence interval) to test the significance of the mediation effects. The results (see Table 3) showed that the total indirect effect of growth mindset on EFL boredom was −0.621, accounting for 78.71% of the total effect (−0.789), whereas the direct effect was −0.168, accounting for 21.29% of the total effect (−0.789), indicating that the mediation effects were significant. In addition, all indirect effects were negative, suggesting that growth mindset reduced EFL boredom by enhancing learners’ AI literacy and AI self-efficacy.

1. Regarding the independent mediating effect of AI literacy, the indirect effect analysis showed that the 95% confidence interval for the mediation coefficient did not include zero (−0.297 ~ −0.177), indicating that the mediation effect was statistically significant. This result confirmed that the indirect pathway “Growth Mindset → AI Literacy → EFL Boredom” was significant, with the indirect effect accounting for 32.70% of the total effect.
2. Regarding the independent mediating effect of AI self-efficacy, the indirect effect analysis showed that the 95% confidence interval for the mediation coefficient did not include zero (−0.371 ~ −0.234), indicating that the mediation effect was statistically significant. This result confirmed that the indirect pathway “Growth Mindset → AI Self-Efficacy → EFL Boredom” was significant, with the indirect effect accounting for 33.59% of the total effect.
3. Regarding the sequential mediating effect of AI literacy and AI self-efficacy, the indirect effect analysis showed that the 95% confidence interval for the mediation coefficient did not include zero (−0.175 ~ −0.069), indicating that the mediation effect was statistically significant. This result confirmed that the indirect pathway “Growth Mindset → AI Literacy → AI Self-Efficacy → EFL Boredom” was significant, with the indirect effect accounting for 12.42% of the total effect.

**Table 3:** Mediation analysis (*N* = 536).

Paths	Effect	95% Boot CI	Standard error	Effect percentage
Growth mindset → AI literacy → EFL boredom	−0.258	[−0.297, −0.177]	0.04	32.70%
Growth mindset → AI self-efficacy → EFL boredom	−0.265	[−0.371, −0.234]	0.05	33.59%
Growth mindset → AI literacy → AI self-efficacy → EFL boredom	−0.098	[−0.175, −0.069]	0.04	12.42%
Total indirect effect	−0.621	[−0.638, −0.572]	0.03	78.71%
Direct effect	−0.168	[−0.255, 0.084]	0.04	21.29%
Total effect	−0.789	[−0.932, −0.644]	0.06	100%

### 4.4 Summary

**Figure 2:**
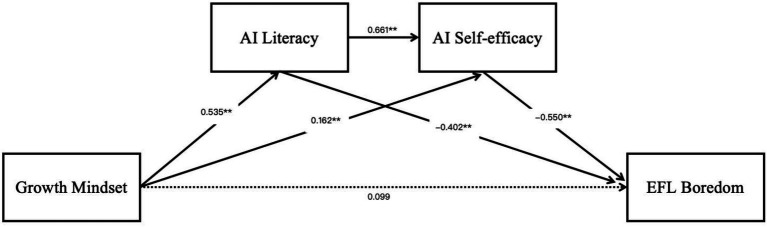
Relationships between growth mindset, AI literacy, AI self-efficacy and EFL boredom.

Based on the data analyses and findings presented in Sections 4.1 to 4.3, the empirical model of this study is illustrated in Figure 2. The results indicate that Hypothesis 1, which proposed that growth mindset would negatively predict EFL boredom, was not supported. In contrast, Hypotheses 2, 3, and 4 were all supported. Specifically, growth mindset positively predicted both AI literacy and AI self-efficacy; AI literacy and AI self-efficacy both negatively predicted EFL boredom; AI literacy positively predicted AI self-efficacy; and AI literacy together with AI self-efficacy exerted a significant sequential mediating effect in the relationship between growth mindset and EFL boredom.

## 5 Discussion

Contrary to Hypothesis 1, this study found that growth mindset did not directly predict EFL boredom in AI-assisted learning contexts surprisingly. This finding differs from previous studies suggesting that growth mindset can effectively reduce negative academic emotions and foster more adaptive emotional experiences in language learning (e.g., Wang et al., 2021; Derakhshan et al., 2022; Zhang et al., 2024). The result indicates that the emotional mechanisms underlying EFL boredom may become more complex in technology-enhanced learning environments. AI-assisted language learning may require learners not only to maintain positive beliefs about the development of language ability but also to interact effectively with AI technologies during learning tasks (Lee and Lee, 2024). Compared with traditional EFL classrooms, AI-mediated learning environments involve additional technological demands, such as understanding AI-generated feedback, evaluating the quality of AI outputs, and using appropriate prompts to accomplish language tasks (Barrot, 2023; Vogt and Flindt, 2023). Therefore, although learners with a growth mindset tend to persist in challenging situations and believe that improvement can be achieved through effort (Dweck, 2006), such beliefs alone may not directly reduce EFL boredom if learners lack sufficient AI-related competencies. From the perspective of CVT (Pekrun, 2006), it posits that achievement emotions are closely associated with learners’ perceived control and task value. In AI-assisted EFL learning contexts, even when learners hold adaptive learning beliefs, they may still experience EFL boredom if they feel uncertain or ineffective in using AI tools (Liu, 2026). In other words, growth mindset may not directly enhance learners’ sense of control over AI-supported learning activities unless it is translated into practical competencies such as AI literacy and AI self-efficacy. Moreover, EFL boredom in AI-assisted environments may partly stem from passive or inefficient interactions with technology. Learners who are unable to critically evaluate AI-generated content or effectively utilize AI tools may become overly dependent on AI systems, thereby reducing their cognitive engagement and active participation in learning tasks (Ma and Chen, 2024). Such experiences may further intensify learners’ sense of disengagement and EFL boredom. Therefore, the influence of growth mindset on EFL boredom in AI-assisted EFL learning may operate primarily through learners’ AI-related competencies rather than through a direct pathway.

However, this study confirmed and supported Hypotheses 2 and 3, demonstrating that both AI literacy and AI self-efficacy significantly mediated the relationship between growth mindset and EFL boredom. This finding suggests that growth mindset does not directly reduce learners’ EFL boredom; rather, it influences emotional experiences by enhancing learners’ AI-related competencies and technological beliefs. These results not only reveal the underlying mechanisms through which growth mindset affects EFL boredom but also further indicate that learners’ technological adaptability has become a crucial factor shaping emotional experiences in AI-assisted EFL learning environments. First, the study found that growth mindset positively predicted AI literacy, but AI literacy negatively predicted EFL boredom, which is consistent with current studies (e.g., Liu, 2026). This suggests that learners with a growth mindset are more willing to actively engage with, explore, and learn AI technologies and are more likely to maintain openness and adaptability when facing challenges in AI-assisted learning (Huang et al., 2025). Growth mindset emphasizes that abilities can be continuously developed through sustained effort and learning (Dweck, 2006). Consequently, such learners are more inclined to experiment with new technologies, adjust learning strategies, and embrace learning challenges. In AI-assisted EFL learning contexts, this positive cognitive orientation facilitates learners’ understanding, evaluation, and effective use of AI tools, thereby fostering higher levels of AI literacy (Liu, 2026). At the same time, higher AI literacy enables learners to use AI tools more skillfully for EFL writing, translation, reading comprehension, and information retrieval tasks, while also helping them effectively evaluate and filter AI-generated content (Wang and Wang, 2025). This not only enhances learners’ sense of task control and interactive engagement during the learning process but also increases the personalization and novelty of learning experiences, thereby reducing EFL boredom caused by mechanical and repetitive learning activities. Therefore, AI literacy serves as an important bridge between growth mindset and EFL boredom.

In addition, this study found that AI self-efficacy significantly mediated the relationship between growth mindset and EFL boredom. Specifically, growth mindset significantly enhanced learners’ AI self-efficacy, whereas AI self-efficacy negatively predicted EFL boredom. From the perspective of SCT (Bandura, 1986, 2003), individuals with stronger confidence in their ability to accomplish tasks are more likely to maintain active behavioral engagement and sustained involvement during learning while experiencing lower levels of negative emotions such as anxiety and frustration. In AI-assisted EFL learning environments, learners are required to interact frequently with AI tools and use them to complete tasks (Derakhshan et al., 2025). In this process, higher levels of AI self-efficacy enhance learners’ sense of control and confidence in operating AI technologies, leading them to believe that they can effectively use AI tools to solve learning problems. Consequently, learners become more willing to actively experiment, sustain engagement, and participate in classroom activities when confronting AI-related learning tasks, thereby reducing uncertainty, anxiety, and helplessness associated with technology use and ultimately alleviating EFL boredom. In contrast, when learners lack AI self-efficacy, even those with relatively strong growth mindsets may still experience considerable technological pressure and negative emotions during AI-assisted learning due to insufficient confidence in their AI-related abilities, which may in turn increase their susceptibility to EFL boredom.

Lastly, this study supported Hypothesis 4, indicating that AI literacy and AI self-efficacy jointly functioned as significant sequential mediators in the relationship between growth mindset and EFL boredom. Specifically, growth mindset was positively associated not only with learners’ AI literacy but also, indirectly, with their AI self-efficacy through AI literacy, which in turn was linked to lower levels of EFL boredom. This finding suggests that AI literacy and AI self-efficacy may not be entirely independent constructs; rather, they appear to exhibit a progressive relationship and intrinsic connection, which is generally consistent with previous research (e.g., Bewersdorff et al., 2025). Learners with a growth mindset may be more willing to actively engage with novel learning tools and approaches, experiment with new learning methods, and maintain greater resilience and openness when encountering difficulties in AI-assisted learning. Such positive learning beliefs may facilitate the accumulation of AI-related knowledge and technological experience, thereby contributing to higher levels of AI literacy (Huang et al., 2025). At the same time, learners with higher levels of AI literacy may demonstrate stronger abilities to understand, operate, and evaluate AI tools, which could further enhance their sense of control and technological confidence in AI-assisted learning and subsequently relate to higher levels of AI self-efficacy. In this regard, AI literacy may provide an important cognitive foundation for the development of AI self-efficacy (Bewersdorff et al., 2025). When learners better understand the operational logic of AI tools and possess the fundamental skills required for AI-assisted learning, they may be more likely to develop positive self-perceptions regarding their ability to effectively use AI to accomplish learning tasks. Therefore, AI literacy may not merely represent a form of technological competence but may also be associated with learners’ confidence in and identification with their own AI-use capabilities. Furthermore, the results indicated that the combined effects of AI literacy and AI self-efficacy were associated with lower levels of EFL boredom (Liu, 2026). For one thing, higher levels of AI literacy may enable learners to use AI tools more efficiently and flexibly in completing learning tasks, thereby potentially enhancing the interactivity and novelty of learning activities. For another, higher AI self-efficacy may reduce learners’ anxiety and uncertainty during AI use while strengthening their learning engagement and willingness to sustain participation (Ren et al., 2026). Consequently, when learners simultaneously possess relatively high levels of AI literacy and AI self-efficacy, they may be more likely to maintain positive learning experiences in AI-assisted EFL learning and report lower levels of boredom (Liu, 2026). Overall, the sequential mediation pathway identified in this study suggests that, in AI-assisted EFL learning environments, the relationship between growth mindset and learning emotions may operate through AI-related competencies and beliefs such as AI literacy and AI self-efficacy, rather than being directly manifested. These findings not only extend the research perspective on growth mindset in the AI era but also further highlight the potential role of AI-related competencies in the formation of emotions in EFL learning.

Admittedly, despite examining the relationships among growth mindset, AI literacy, AI self-efficacy, and EFL boredom in the context of AI-assisted EFL learning and employing a time-lagged design to strengthen the temporal sequencing among variables, this study still has several limitations. First, the sample source was relatively limited, as only university students from two higher education institutions in eastern China were recruited through convenience sampling. Therefore, the representativeness of the sample remains limited, and the applicability of the findings across different regions, institutional types, and cultural contexts requires further verification. Second, although this study adopted a time-lagged design, which to some extent strengthened the temporal order among variables, it still cannot fully establish strict causal relationships. Moreover, the relatively limited time span makes it difficult to comprehensively capture the long-term dynamic changes of emotional and psychological variables in AI-assisted EFL learning processes. Thirdly, although the present study employed a general growth mindset scale to capture learners’ broader beliefs about ability development and technological adaptation in AI-assisted learning environments, a language-specific mindset scale may have provided more context-sensitive insights into learners’ beliefs in EFL learning contexts. Future studies are encouraged to compare the effects of general and domain-specific mindset measures in AI-assisted EFL learning research. Finally, this study did not further differentiate among various types of AI tools and their specific usage patterns. Different AI technologies may vary substantially in terms of interaction modes, feedback mechanisms, and learning-support functions, which may in turn exert different influences on learners’ emotional experiences.

## 6 Implications

At the theoretical level, this study extends the application of both SCT and CVT to AI-assisted EFL learning contexts. The findings highlight AI literacy and AI self-efficacy as important psychological resources in AI-assisted EFL learning. Consistent with recent studies suggesting that AI-mediated learning environments can facilitate learners’ technological adaptation, confidence, and self-efficacy through human-and-AI interactions (e.g., Guan et al., 2024; Zhang et al., 2026), the present study further supports the applicability of SCT in explaining the role of AI-related competencies and efficacy beliefs in technology-enhanced learning. Meanwhile, by identifying AI literacy and AI self-efficacy as important antecedents of EFL boredom, this study broadens the applicability of CVT and suggests that AI-related competencies and beliefs may play an important role in shaping learners’ emotional experiences in AI-assisted learning environments. This finding also aligns with recent CVT-based research in AI-mediated learning (e.g., Wang et al., 2026), which has highlighted the importance of control- and value-related factors in shaping learners’ achievement emotions and engagement in AI-supported learning contexts. More importantly, this study contributes to the integration of SCT and CVT in AI-assisted EFL learning research. While SCT helps explain the development of AI-related competencies and efficacy beliefs, CVT provides insights into how these factors are associated with learners’ emotional experiences. By linking these two theoretical perspectives, the present study offers a more comprehensive understanding of the psychological mechanisms underlying EFL boredom in AI-assisted learning contexts.

At the practical level, the findings imply that EFL instruction in AI-assisted environments should move beyond the cultivation of positive learning beliefs alone and place greater emphasis on learners’ AI-related competencies and technological confidence. Accordingly, EFL teachers are encouraged to integrate growth mindset cultivation with AI literacy education and AI tool training through activities such as AI-assisted writing, AI-based evaluation tasks, and critical analysis of AI-generated content. In addition, scaffolded instruction, peer collaboration, and timely feedback may help learners gradually build confidence in using AI tools, thereby enhancing engagement and reducing EFL boredom. Universities should also provide systematic AI training resources and technical support to facilitate learners’ adaptation to AI-assisted learning environments.

## 7 Conclusion

This study, situated within the context of AI-assisted EFL learning, employed a time-lagged research design to systematically examine the relationships among growth mindset, AI literacy, AI self-efficacy, and EFL boredom, and further constructed a sequential mediation model. The findings revealed that growth mindset positively predicted both AI literacy and AI self-efficacy; AI literacy and AI self-efficacy both negatively predicted EFL boredom; and AI literacy further promoted the development of AI self-efficacy. In addition, both AI literacy and AI self-efficacy exerted significant independent mediating effects as well as a sequential mediating effect in the relationship between growth mindset and EFL boredom. Notably, it found that growth mindset did not directly predict EFL boredom. This finding suggests that, in AI-assisted EFL learning environments, learners’ positive beliefs about the malleability of ability are insufficient to directly alleviate negative emotions; rather, their influence must be realized through AI-related competencies and technological beliefs. In other words, emotional experiences in EFL learning in the AI era are shaped not only by traditional psychological factors but also increasingly constrained by learners’ AI competencies and levels of technological adaptability.

## Data Availability

The raw data supporting the conclusions of this article will be made available by the authors, without undue reservation.
